# Prediction of infliximab and anti-drug antibody concentrations in patients with inflammatory bowel disease using machine learning models with real-world data from a prospective cohort study

**DOI:** 10.3389/fphar.2026.1731193

**Published:** 2026-01-28

**Authors:** Minjung Kim, Joo Hye Song, Sung Noh Hong, Myeong Gyu Kim, Eun Ran Kim, Dong Kyung Chang, Young-Ho Kim

**Affiliations:** 1 College of Pharmacy, Graduate School of Pharmaceutical Sciences, Ewha Womans University, Seoul, Republic of Korea; 2 Department of Internal Medicine, Konkuk University Medical Center, Konkuk University School of Medicine, Seoul, Republic of Korea; 3 Department of Medicine, Samsung Medical Center, Sungkyunkwan University School of Medicine, Seoul, Republic of Korea

**Keywords:** anti drug antibodies (ADA), concentration, inflammatory bowel disease, infliximab, machine learning, prediction model

## Abstract

**Background:**

Although population pharmacokinetic models are the standard approach for identifying inter-individual variability and optimizing infliximab concentration, their development and validation are complex and time-consuming. Therefore, this study aimed to develop and evaluate machine learning (ML) models to predict infliximab and anti-drug antibody (ADA) concentrations in patients with inflammatory bowel disease (IBD) receiving maintenance infliximab therapy.

**Methods:**

A total of 1,806 infliximab and ADA concentration measurements were prospectively collected from 149 IBD patients. Recurrent neural networks (RNN)-based models, including long short-term memory (LSTM) and gated recurrent unit (GRU) architectures, as well as regression-based models such as Elastic Net, Support Vector Regression, Random Forest (RF), and extreme gradient boosting (XGBoost), were developed. Recursive multi-step prediction was applied to evaluate short-term forecasting performance.

**Results:**

RF outperformed in predicting infliximab concentrations, and XGBoost yielded the best performance in predicting ADA levels (2-fold accuracy, 86.67% and 96.67%, respectively). The infliximab prediction model maintained acceptable accuracy up to two recursive predictions steps but exhibited a notable performance decline at the third step. In contrast, the ADA model showed robust performance across all three recursive steps, maintaining 2-fold accuracy exceeding 96%.

**Conclusion:**

ML models were developed to predict infliximab and ADA concentrations, with RF and XGBoost showing the best performance for infliximab and ADA prediction, respectively. The ADA model demonstrated stable multi-step forecasting capability. These models may support individualized dosing strategies and reduce the need for frequent therapeutic drug monitoring in clinical practice.

## 1 Introduction

Inflammatory bowel disease (IBD), encompassing Crohn’s disease (CD) and ulcerative colitis (UC), is a chronic, relapsing inflammatory disorder of the gastrointestinal tract (Chauhan and Rieder, 2025; Dias et al., 2024). Infliximab, a monoclonal antibody targeting tumor necrosis factor-α (TNF-α), effectively induces and maintains remission in IBD (Papamichael et al., 2019). However, up to 30% of patients exhibit primary non-response, and many initial responders eventually experience loss of response (Papamichael et al., 2019). Substantial inter-individual variability in infliximab pharmacokinetics and the development of anti-drug antibodies (ADA) can compromise therapeutic efficacy, leading to subtherapeutic drug levels and diminished clinical benefit (Fasanmade et al., 2009; Wang et al., 2025; Baert et al., 2003; Brun et al., 2024).

Therapeutic drug monitoring (TDM) of infliximab trough concentrations and ADA levels is increasingly recognized as essential for optimizing dosing strategies (Hu et al., 2024; Roblin et al., 2024; Mitchell et al., 2016). In clinical settings where only sparse sampling is feasible, population pharmacokinetic modeling is the standard method used to characterize variability in drug concentrations among individuals (Ette and Williams, 2004). Despite its utility, this approach relies on predefined structural assumptions such as one- or two-compartment models, and requires the integration of multiple covariates and their interactions (Byon et al., 2013). As a result, model development becomes complex, and iterative processes for model validation and refinement are time-consuming. Moreover, because real-world data often provide only trough concentrations, it becomes difficult to estimate parameters related to the absorption phase, which are often fixed rather than modeled.

Recent advances in machine learning (ML) offer a complementary, data-driven paradigm for individualized pharmacokinetic prediction. Deep learning architectures such as recurrent neural networks (RNNs), particularly long short-term memory (LSTM) and gated recurrent unit (GRU) models, are well-suited for modeling longitudinal drug concentration data with temporal dependencies (Hochreiter and Schmidhuber, 1997; Cho et al., 2014). The LSTM is a type of RNN designed for sequential data (Hochreiter and Schmidhuber, 1997). Their architecture includes memory cells and gates to selectively retain or discard information, making them ideal for capturing long-term dependencies in time series data (Hochreiter and Schmidhuber, 1997). The GRU simplifies the LSTM architecture by combining the forget and input gates into one update gate, enabling faster training (Cho et al., 2014). When combined with patient characteristics, these models can provide more nuanced and personalized predictions of drug levels over time. In addition to deep learning, conventional ML algorithms, including Elastic Net regression, Support Vector Regression (SVR), Random Forest (RF), and extreme gradient boosting (XGBoost), have demonstrated effectiveness in biomedical prediction tasks and offer interpretable, robust alternatives that complement neural network-based models (Zou and Hastie, 2005; Cortes and Vapnik, 1995; Ho, 2002; Chen and Guestrin, 2016). Therefore, these ML approaches can predict infliximab and ADA concentrations at specific time points using repeated measurements without explicitly specifying pharmacokinetic structures. Moreover, explainable artificial intelligence techniques like SHAP (SHapley Additive exPlanations) enhance transparency by quantifying the contribution of each input feature to the model output (Lundberg and Lee, 2017).

Although various ML models have been employed to predict treatment response in IBD patients receiving infliximab (Qiu et al., 2025; Morilla et al., 2019; Con et al., 2021), few studies have directly focused on estimating infliximab concentrations (Irie et al., 2025a; Irie et al., 2025b). Meanwhile, pharmacokinetic prediction using ML has been extensively explored for other biologics and small-molecule drugs, highlighting the feasibility and clinical relevance of data-driven approaches to personalized dosing. For instance, XGBoost models have been utilized to optimize vancomycin dosing by incorporating second-order variable interactions and high-dimensional clinical data through comprehensive feature engineering (Huang et al., 2021). Likewise, an LSTM model outperformed conventional population pharmacokinetic models in predicting valproic acid concentrations in elderly patients with epilepsy (Soeorg et al., 2022). Similarly, a GRU model demonstrated superior accuracy in predicting the early distribution kinetics of propofol in both morbidly obese and lean individuals (Ingrande et al., 2020).

This study aimed to develop ML models capable of accurately predicting infliximab and ADA concentrations in IBD patients with infliximab maintenance therapy, and evaluate their predictive accuracy and interpretability for supporting individualized therapy adjustments in real-world clinical settings.

## 2 Materials and methods

### 2.1 Study population

This study utilized real-world data prospectively collected from IBD patients at Samsung Medical Center in Seoul, Korea, between February 2020 and December 2022. The inclusion and exclusion criteria were identical to those described in our previous study (Song et al., 2025). In brief, patients with CD or UC receiving maintenance intravenous (IV) infliximab who consented to proactive TDM, including trough infliximab and ADA titer measurements, were enrolled. Additionally, the exclusion criteria in this study were expanded to include individuals who switched to other biologics or subcutaneous infliximab within 6 months of enrollment. For patients who switched to subcutaneous infliximab after 6 months of enrollment, only data collected prior to the switch were included in the analysis, and all post-switch data were excluded. The study was approved by the Institutional Review Board of Samsung Medical Center (approval number: 2019-05-079). The study protocol adhered to the ethical guidelines of the 1975 Declaration of Helsinki, as reflected in a prior approval by the institution’s human research committee.

### 2.2 Infliximab maintenance therapy with dose optimization

Patients received induction therapy with 5 mg/kg IV infliximab (Remicade^®^, Janssen Pharmaceutica, Beerse, Belgium; Remsima^®^, Celltrion, Incheon, Korea; and Relamoce^®^, Samsung bioepis, Incheon, Korea) at 0, 2, and 6 weeks, followed by maintenance therapy with 5 mg/kg every 8 weeks. The dosage of infliximab was adjusted based on the trough concentrations. Patients with sub-therapeutic levels (<3 μg/mL) or loss of response were considered for dose escalation to 10 mg/kg or switching to other biologics.

### 2.3 Data collection

Data collection process were identical to those described in our previous study (Song et al., 2025). In brief, at each outpatient visit, demographic information (age, sex, body weight, and height), laboratory findings (white blood cell count [WBC], albumin, and C-reactive protein [CRP] levels), and pharmacokinetic (PK) data—including serum concentrations of infliximab and ADA, as well as dosage and administration interval (i.e., the time from administration to sampling before the next dose)—were collected. Clinical characteristics were also recorded, including Montreal classification (location, behavior, and perianal modifier for CD; disease extent for UC), smoking and surgical history, indication and type of infliximab, and concomitant use of immunomodulators. At enrollment, residual disease burden was assessed. In CD, this included mucosal healing or endoscopic remission; in UC, endoscopic remission. Transmural healing in CD was defined as a global simplified magnetic resonance index of activity (sMaRIA) score of 0. The sMaRIA was calculated using the formula: [1×wall thickness (>3 mm)] + [1×mural oedema (hyperintense signal on fat-saturated T2-weighted images)] + [1×fat stranding (increased T2-weighted signal intensity in mesenteric fat adjacent to bowel loops due to oedema/fluid in perienteric fat)] + [2×ulcers] (Ordás et al., 2019). The global sMaRIA score was obtained by summing segmental scores from the rectum, sigmoid colon, descending colon, transverse colon, ascending colon, and ileum (Roseira et al., 2020). If magnetic resonance enterography was not available, endoscopic remission was assessed using the Simple Endoscopic Score for Crohn’s Disease (SES-CD), with a score of 0 indicating complete remission (Klenske et al., 2019). In UC, endoscopic remission was defined as a Mayo endoscopic subscore of 0.

A total of 10 mL of whole blood was collected in ethylene-diamine-tetraacetic acid tubes immediately before infliximab infusion, and the serum was isolated using centrifugation. Serum infliximab concentrations were measured using the Remsima kit (Immundiagnostik AG, Bensheim, Germany), following the manufacturer’s instructions (Song et al., 2022; Remsima, 2018). The concentration of free infliximab, was measured with an Enzyme-Linked Immunosorbent Assay (ELISA) reader. And total ADA levels, including both free and infliximab-bound antibodies, were measured using an ELISA.

### 2.4 RNN-based model development

**FIGURE 1:**
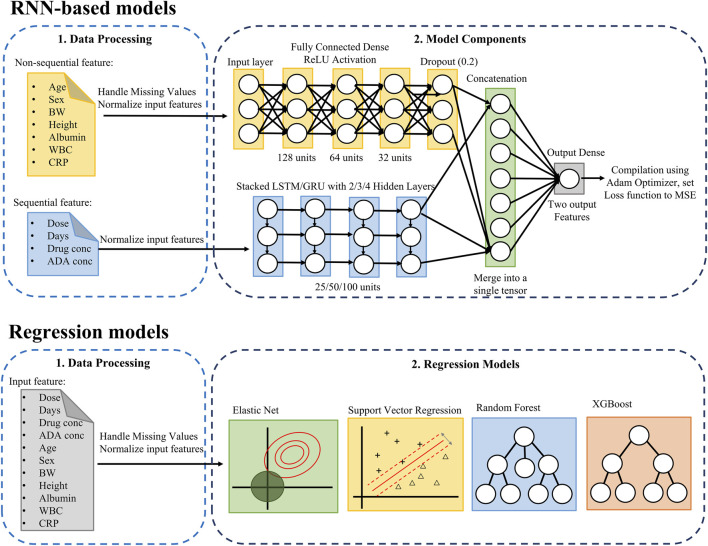
Pipeline for predicting infliximab and anti-drug antibody concentrations using RNN-based and regression models. The upper RNN-based models section combines non-sequential and sequential data to predict infliximab and ADA concentrations, while the Regression Model section applies regression models to all features. ADA, anti-drug antibody; BW, Body Weight; CRP, C-Reactive Protein; GRU, Gated Recurrent Unit; MSE, Mean Squared Error; LSTM, Long Short-Term Memory; ReLU, Rectified Linear Unit; RNN, Recurrent Neural Network; WBC, White Blood Cell.

The model was designed to incorporate both sequential features and non-sequential covariates. Sequential features included infliximab and ADA concentrations, the time interval between drug administration and serum sampling, and infliximab dosage, which were organized as ordered time-windows to capture temporal dependencies (Figure 1). To preprocess the temporal data for use in LSTM and GRU models, a sliding window approach was applied. A fixed window of three prior time steps was used to predict the subsequent time step, formulating the task as a sequence-to-one prediction problem. Specifically, for each patient, infliximab and ADA concentrations together with other sequential features from three consecutive time points, were used to predict concentrations at the next time point. Supplementary Figure S1 illustrates the construction of the training and testing datasets using this approach. Non-sequential covariates included age, sex, body weight, height, albumin, WBC, and CRP levels. These covariates were incorporated as visit-level inputs at each prediction time point, rather than being modeled as longitudinal sequences. Missing values were imputed using the temporally nearest available measurements within each patient’s dataset. The dataset was split into training and testing sets with an 80:20 ratio. Min–Max normalization was fitted on the training set and applied to the testing set.

The model architecture integrates a deep neural network (DNN) for non-sequential covariates and a multi-layer LSTM/GRU network for sequential features. The non-sequential covariates were processed through a DNN comprising three fully connected layers with 128, 64, and 32 hidden units, respectively, each employing the ReLU activation function. Dropout layers were included to mitigate overfitting. Sequential features were modeled using LSTM/GRU networks with 2, 3, 4 layers, and each configuration was evaluated with hidden sizes of 25, 50, and 100 units. Among these, the 2-layer network with 100 hidden units demonstrated the best performance. The outputs from the DNN and LSTM/GRU components were concatenated and passed through a final fully connected layer. The model was optimized using the Adam optimizer with mean squared error as the loss function. The output layer comprised two units, jointly representing infliximab and ADA concentrations as a two-dimensional vector.

### 2.5 Conventional regression models development

Several conventional regression models were implemented, including Elastic Net, SVR, RF, and XGBoost (Figure 1). The input features included previously measured infliximab concentrations, ADA concentrations, the time interval between drug administration and serum sampling, the infliximab dose ratio (defined as the ratio of the most recent to the prior dose), age, sex, body weight, height, albumin, WBC, and CRP levels. A patient-level split was used to divide the dataset into training and testing sets in an 80:20 ratio. All features were normalized using Min-Max scaling.

Hyperparameter tuning was conducted on the training set using grid search with 5-fold cross-validation. The performance metric used for optimization was the negative mean squared error. The specific hyperparameter values tested for each model are summarized in the Supplementary Table S1. Feature importance was further evaluated using SHAP values. Features with consistently low SHAP values were iteratively removed, as long as model performance did not significantly deteriorate. This approach allowed for effective dimensionality reduction without compromising prediction accuracy.

### 2.6 Model evaluation

Model performance was assessed using mean squared error (MSE). In addition, extended evaluation metrics commonly adopted in pharmacokinetic modeling were used, including the percentage of predictions with relative errors within 20% (F20) and 30% (F30) of the actual value, and the 2-fold accuracy (Mao et al., 2022). The model’s predictive performance was considered satisfactory if it met the following criteria: F20 > 35% and F30 > 50% (Mao et al., 2018). The 2-fold accuracy reflects the proportion of predictions falling within two-fold (i.e., between 0.5× and 2× the actual value), a widely accepted criterion in pharmacokinetic prediction tasks. The mathematical definitions of these evaluation metrics are as follows:

$$\text{Mean squared error }\left(\text{MSE}\right)=\left(1/n\right)\times \Sigma {\left(y_{i}-{ŷ}_{i}\right)}^{2}$$

$$F20=\left(\text{Number of samples with }\left|y_{i}-{ŷ}_{i}\right|/\;yi\leq 0.2\right)\;/\;n\times 100\%$$

$$F30=\left(\text{Number of samples with }\left|y_{i}-{ŷ}_{i}\right|/\;yi\leq 0.3\right)\;/\;n\times 100\%$$

$$2-\text{fold accuracy}=\left(\text{Number of samples with }0.5\times y_{i}\leq {ŷ}_{i}\leq 2\times yi\right)\;/\;n\times 100\%$$

Where, n denotes the number of samples, y_i_ and ŷ_i_ represent the true and predicted values, respectively.

### 2.7 Recursive prediction for future time points

To assess the model’s ability to predict future values beyond the one-step ahead prediction, a multi-step forecasting approach was applied. Starting from the current input data, the model first generated predictions for infliximab and ADA concentrations at the next time point (Step 1). The Step 1 predictions were subsequently used as inputs to generate predictions for the second future time point (Step 2), and the same recursive process was repeated to obtain predictions for the third future time point (Step 3). At each recursive step (i.e., Step 2 and 3), the predicted infliximab and ADA concentrations from the previous step were recursively incorporated into the sequential input to generate predictions for later time points. All other time-varying covariates (e.g., dosing information, sampling intervals, albumin, WBC, and CRP) were updated using the values corresponding to each respective prediction time point during evaluation. This recursive procedure approximates model performance in multi-step forecasting scenarios where true future concentration measurements are unavailable. Prediction performance was evaluated at each step using performance metrics such as MSE, F20, F30, and 2-fold accuracy.

## 3 Results

### 3.1 Characteristics of the study population

A total of 187 IBD patients were eligible for this study. Thirty-eight patients were excluded for the following reasons: withdrew consent (n = 1), drug holiday >4 months (n = 5), or switching to other biologics including subcutaneous infliximab within 6 months after enrollment (n = 32). Finally, 149 patients (122 CD and 27 UC) were included. Baseline characteristics of the study population are summarized in Table 1. The median age of enrolled patients was 36 years (interquartile range [IQR], 30–45) and 75.2% of patients were male. 18.6% of CD patients showed transmural healing or endoscopic remission, and 55.6% of UC patients showed endoscopic remission at the enrollment. Less than half of the patients used concomitant immunomodulator.

**TABLE 1:** Characteristics of enrolled patients.

Characteristics	Value
Demographics	(N = 149)
Age (year), median (IQR)	36 (30, 45)
Male, n (%)	112 (75.2)
Body weight (kg), median (IQR)	66.1 (55.4, 73.9)
Height (cm), median (IQR)	170 (163, 175)
Diagnosis	
Crohn’s disease, n (%)	122 (81.9)
Ulcerative colitis, n (%)	27 (18.1)
Current smoker, n (%)	23 (15.4)
Previous intestinal surgery, n (%)	43 (28.9)
Age at diagnosis	
<16 years, n (%)	10 (6.7)
17–40, n (%)	122 (81.9)
>40, n (%)	17 (11.4)
Montreal classification	
CD: Location	
Ileal, n (%)	29 (23.8)
Colonic, n (%)	11 (9.0)
Ileocolonic, n (%)	82 (67.2)
Upper GI, n (%)	22 (18.0)
CD: Behavior	
Inflammatory, n (%)	47 (38.5)
Stricturing, n (%)	34 (27.9)
Penetrating, n (%)	41 (33.6)
CD: Perianal disease, n (%)	67 (54.9)
UC: Disease extent	
Proctitis, n (%)	1 (3.7)
Left side, n (%)	11 (40.7)
Extensive, n (%)	15 (55.6)
Remnant disease burden at the enrollment	
CD	
Transmural healing (global sMaRIA score = 0)	16/86 (18.6%)
Endoscopic remission (SES-CS = 0)	8/33 (24.2%)
UC	
Endoscopic remission (MES = 0)	15/27 (55.6%)
Drug therapy	
Type of infliximab	
Remicade^®^	102 (68.5)
Remsima^®^	37 (24.8)
Remaloce^®^	10 (6.7)
Indications for infliximab	
Fistulizing, n (%)	15 (10.1)
Active luminal, n (%)	134 (89.9)
Concomitant immunomodulators, n (%)	72 (48.3)
Dose intensification (10 mg/kg) at enrollment, n (%)	36 (24.2)
Laboratory measurements	(N = 1,806)
White blood cell (×10^9^/L), median (IQR)	5.7 (4.7, 6.8)
Albumin (g/dL), median (IQR)	4.4 (4.2, 4.6)
C-reactive protein (mg/dL), median (IQR)	0.06 (0.06, 0.13)
Infliximab and ADA-related variables	(N = 1,806)
Infliximab dose (mg), median (IQR)	400 (300, 600)
Infliximab concentration (ng/mL), median (IQR)	4.23 (2.56, 6.32)
ADA concentration (ng/mL), median (IQR)	9.33 (7.60, 11.5)
Time from infliximab administration to sampling (days), median (IQR)	56 (55, 57)

ADA, anti-drug antibody; CD, Crohn’s disease; IQR, interquartile range; MES, mayo endoscopic score; SES-CD, Simple Endoscopic Score for Crohn’s Disease; sMaRIA, simplified magnetic resonance index of activity; UC, ulcerative colitis.

At the time of enrollment, 113 patients treated with IV infliximab at a dose of 5 mg/kg. Among them, 63.7% (72/113) maintained the 5 mg/kg dose, while 36.3% (41/113) escalated to 10 mg/kg. Ten patients were subsequently switched to subcutaneous infliximab, and one patient transitioned to a different biologic agent. Additionally, 36 patients (24.2%) were already receiving 10 mg/kg IV infliximab at enrollment, of whom 13 were later switched to subcutaneous infliximab.

In total, 1,806 observations were available for infliximab and ADA concentrations. The median dosage of infliximab was 400 mg (IQR, 300–600), and time interval between drug administration and serum sampling was consistently around 56 days (IQR, 55–57). The median serum infliximab concentration of 4.23 μg/mL (IQR, 2.56–6.32) and ADA concentration of 9.33 ng/mL (IQR, 7.60–11.5).

### 3.2 Model comparison summary

The predictive performance of RNN-based models (LSTM and GRU) and conventional ML models (Elastic Net, SVR, RF, and XGBoost) was evaluated using the testing dataset. Table 2 summarizes the model performance across four evaluation metrics: MSE, F20 and F30, and 2-fold accuracy. For infliximab concentration prediction, RF demonstrated the best overall performance with the lowest MSE (3.370) and the highest F30 (60.00%). It also achieved a high F20 of 43.33% and a 2-fold accuracy of 86.67%. XGBoost also performed competitively, particularly in terms of 2-fold accuracy (90.00%), albeit with a slightly higher MSE (5.494). Among the RNN-based models, GRU outperformed LSTM across all metrics, achieving a lower MSE (12.272 vs. 12.634), higher F20 (39.56% vs. 37.73%), and higher 2-fold accuracy (83.15% vs. 81.68%). However, both models were generally outperformed by RF and XGBoost in terms of error metrics and accuracy thresholds. Elastic Net and SVR showed moderate performance, with MSE values of 6.941 and 6.836, respectively. Despite having lower 2-fold accuracy compared to tree-based models, SVR achieved an F30 of 54.55%, which was comparable to that of GRU.

**TABLE 2:** Predictive performance of machine learning models for infliximab and ADA concentrations in the testing dataset.

Performance metrics	LSTM	GRU	Elastic net	SVR	RF	XGBoost
Outcome 1 (Infliximab)						
MSE	12.634	12.272	6.941	6.836	**3.370**	5.494
F_20_ (%)	37.73	39.56	27.27	31.82	**43.33**	40.00
F_30_ (%)	55.31	54.58	40.91	54.55	**60.00**	53.33
2-fold accuracy (%)	81.68	83.15	86.36	81.82	**86.67**	90.00
Outcome 2 (ADA)						
MSE	171.395	165.568	19.078	21.210	24.015	**18.046**
F_20_ (%)	57.88	55.68	59.09	50.00	56.67	**53.33**
F_30_ (%)	72.89	72.53	72.73	77.27	76.67	**73.33**
2-fold accuracy (%)	95.97	94.87	95.45	95.45	96.67	**96.67**

The best-performing model based on the lowest MSE for each outcome is highlighted in bold.

ADA, anti-drug antibody; F_20_ and F_30_, percentages of predictions with relative errors within 20% and 30%, respectively; GRU, gated recurrent unit; LSTM, long short-term memory; MSE, mean squared error; RF, random forest; SVR, support vector regression, XGBoost, extreme gradient boosting.

In predicting ADA concentrations, the performance patterns differed from infliximab. XGBoost achieved the best overall performance with the lowest MSE (18.046) and high values for all accuracy metrics (F20: 53.33%, F30: 73.33%, 2-fold accuracy: 96.67%). Elastic Net emerged as a particularly strong performer for ADA, with the highest F20 (59.09%) and a very low MSE (19.078), slightly higher than XGBoost. RF and SVR also performed well, with comparable 2-fold accuracy (both 95.45%–96.67%) and F30 values above 75%. The deep learning models showed the highest MSE values for ADA prediction, with 171.395 (LSTM) and 165.568 (GRU), substantially larger than those of the conventional models. However, they maintained reasonable F20 (LSTM: 57.88%, GRU: 55.68%) and 2-fold accuracy (LSTM: 95.97%, GRU: 94.87%).

### 3.3 Recursive forecasting results (multi-step prediction)

The final predictive models for infliximab and ADA were based on RF and XGBoost, respectively. Feature selection was performed manually in an iterative manner based on SHAP values. Specifically, features with the lowest SHAP contributions were sequentially removed, and model performance was reassessed after each removal. The process was stopped when further feature removal resulted in decreased performance. Consequently, sex was excluded from the final infliximab model, whereas sex, dose ratio, and age were excluded from the final ADA model. Table 3 summarizes the performance metrics of these final predictive models across all prediction steps.

**TABLE 3:** Predictive performance in recursive multi-step forecasting of infliximab and ADA concentrations.

Performance metrics	5-fold CV	Testing (step 1)^a^	Testing (step 2)^a^	Testing (step 3)^a^
Outcome 1 (Infliximab)^b^				
MSE	6.642	3.370	5.657	6.748
F_20_ (%)	42.06	43.33	41.38	14.29
F_30_ (%)	57.49	60.00	62.07	28.57
2-fold accuracy (%)	87.15	86.67	82.76	92.86
Outcome 2 (ADA)^b^				
MSE	71.912	14.397	27.508	33.223
F_20_ (%)	58.31	56.67	62.07	57.14
F_30_ (%)	73.83	83.33	72.41	71.43
2-fold accuracy (%)	95.96	96.67	100	96.43

^a^ Step 1 refers to the first prediction using the current input data. The predicted values were then recursively used to generate Step 2 and Step 3 predictions, simulating multi-step forecasting in the absence of actual future observations.

^b^ Outcome 1 predictions were generated using the random forest (RF) model, excluding the variable sex from the input features. Outcome 2 predictions were obtained using the extreme gradient boosting (XGBoost) model, with sex, dose ratio, and age removed from the feature set.

ADA, anti-drug antibody; CV, cross-validation; F_20_ and F_30_, percentages of predictions with relative errors within 20% and 30%, respectively; MSE, mean squared error.

For infliximab concentration prediction at Step 1, the model achieved an MSE of 3.370, F20 of 43.33%, and F30 of 60.00%, with a 2-fold accuracy of 86.67%. In recursive predictions, in which previously predicted values were used to forecast subsequent time points, model performance gradually declined. At Step 2, the MSE increased to 5.657 with an F30 of 62.07% and a slight decrease in F20% to 41.38%. By Step 3, predictive performance markedly deteriorated in terms of relative accuracy (F20: 14.29%, F30: 28.57%), although 2-fold accuracy remained relatively high at 92.86%. Based on predefined thresholds for satisfactory performance (F20 > 35% and F30 > 50%), the model met the criteria up to Step 2 but failed to maintain adequate accuracy at Step 3.

**FIGURE 2:**
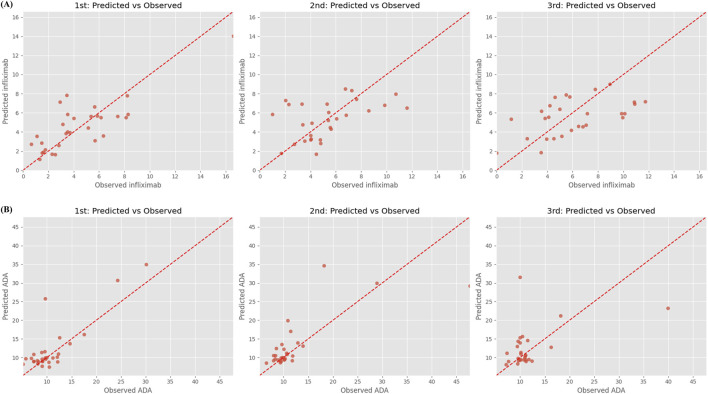
Predicted vs. observed concentrations of **(A)** infliximab and **(B)** anti-drug antibody over three recursive forecasting steps. Step 1 (left), Step 2 (middle), and Step 3 (right) represent predictions made for successive future time points using a recursive multi-step forecasting approach. Each subplot shows predicted concentrations (y-axis) against observed concentrations (x-axis), with the red dashed line indicating the ideal 1:1 agreement. Anti-drug antibody, ADA.

Figure 2 illustrates the predicted versus observed concentrations of infliximab and ADA across the three recursive prediction steps. Consistent with the quantitative results in Table 3, prediction accuracy for infliximab concentrations visibly declines with each recursive step, particularly beyond Step 2. Supplementary Figure S2 presents observed and predicted trajectories of infliximab and ADA levels in 15 randomly selected patients from the test set, highlighting individual-level prediction patterns.

The XGBoost model for ADA prediction demonstrated strong performance in the testing phase. In Step 1, the model achieved a low MSE of 14.397, an F30 of 83.33%, and a high 2-fold accuracy of 96.67%. Recursive prediction performance remained stable across Step 2 and Step 3. Although MSE increased progressively to 27.508 and 33.223, F20 and F30 remained within acceptable ranges (62.07%–57.14% and 72.41%–71.43%, respectively). Notably, the model achieved 100% 2-fold accuracy at Step 2 and maintained 96.43% at Step 3, indicating robust generalizability for near-term ADA forecasting even in the absence of real-time observations.

### 3.4 SHAP-based feature importance analysis

**FIGURE 3:**
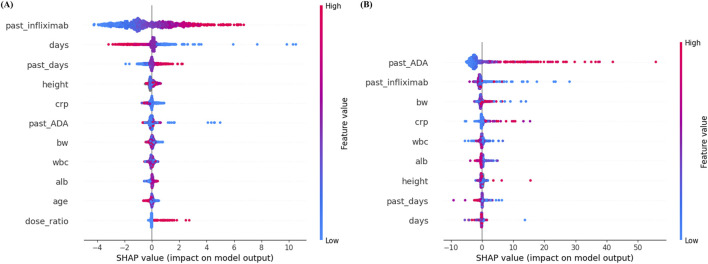
SHAP summary plots for **(A)** infliximab and **(B)** anti-drug antibody prediction models. Each dot represents a SHAP value for an individual sample, with color indicating the original feature value (red = high, blue = low). Features are ranked by mean absolute SHAP value, with higher values indicating greater importance in the model’s predictions. ADA, anti-drug antibody; alb, albumin; BW, body weight; CRP, C-reactive protein; SHAP, SHapley Additive exPlanations; WBC, white blood cell.

The final feature sets are visualized in Figure 3, which presents the distributions of SHAP values for features retained in the final models. For infliximab prediction, past infliximab concentration was the most influential feature, followed by days since last administration and past_days (the interval from prior dosing to the previous sample). Higher past infliximab concentrations were strongly associated with increased predicted levels. Other features, such as height, CRP, and past ADA, showed moderate contributions, while dose ratio and age contributed minimally to the prediction.

In contrast, for ADA prediction, past ADA concentration ranked as the most important predictor, followed by past infliximab concentration and body weight. High past ADA values consistently resulted in higher predicted ADA levels, indicating a strong positive influence on model output. Features such as CRP, WBC, albumin, and height exhibited relatively minor contributions.

## 4 Discussion

To the best of our knowledge, this is the first study to develop ML models for predicting both infliximab and ADA concentrations, and to evaluate their recursive multi-step predictive performance using real-world clinical data. Unlike prior ML studies on infliximab that primarily focused on single-step concentration prediction, this study assessed recursive multi-step forecasting and revealed distinct performance patterns between infliximab and ADA prediction tasks. Using prospectively collected real-world data, ML models based on both RNN and conventional algorithms were developed for IBD patients receiving maintenance infliximab therapy. Overall, tree-based models consistently outperformed RNN-based models, with RF showing the best performance for infliximab prediction (MSE, 3.370; F30, 60.00%; 2-fold accuracy, 86.67%) and XGBoost demonstrating the highest accuracy for ADA prediction (MSE, 18.046; F30, 73.33%; 2-fold accuracy, 96.67%). In recursive multi-step forecasting, the infliximab model maintained acceptable predictive performance up to two steps, whereas the ADA model demonstrated robust performance across all three recursive steps.

Several recent studies have applied ML approaches to predict infliximab concentrations. For example, Irie et al. developed an initial XGBoost model trained on a large synthetic dataset of infliximab concentrations (n = 560,000) generated from an established pediatric population pharmacokinetic model (Irie et al., 2025a). Prediction errors from this model were assessed using real-world data comprising 292 plasma infliximab concentration measurements from 93 pediatric and young adult patients with CD. Subsequently, a second XGBoost model incorporating clinical features including predicted infliximab concentrations, cumulative dose, and dosing interval duration was used to correct these prediction errors. The ensemble model demonstrated improved performance, achieving a 5-fold cross-validated root mean squared error (RMSE) of 4.30 ± 1.09 μg/mL, which was comparable to that of a Bayesian pharmacokinetic approach (RMSE, 4.81 μg/mL) (Irie et al., 2025a). In a subsequent study, the same group proposed a hybrid population pharmacokinetic–ML framework in which ML algorithms were used to adjust prediction errors between Bayesian-estimated and observed infliximab concentrations (Irie et al., 2025b). Among the evaluated models, XGBoost incorporating demographic variables, observed infliximab and ADA concentrations, Bayesian-estimated PK parameters, dosing history, and laboratory markers (albumin, erythrocyte sedimentation rate, and neutrophil CD64) demonstrated the best performance, achieving an RMSE of 3.78 ± 0.85 μg/mL, compared with an RMSE of 4.8 μg/mL for Bayesian estimation alone (Irie et al., 2025b). The predictive performance observed in the present study was within a comparable range to that reported in previous ML-based infliximab studies, despite differences in study populations, modeling frameworks, and prediction tasks.

Traditional population pharmacokinetic modeling is based on biologically interpretable parameters and prior mechanistic assumptions, enabling physiologically meaningful interpretation of drug distribution and clearance. However, such model development follows a structured, iterative, and largely manual process that requires substantial time and expertise and may introduce bias if model assumptions are misspecified (Byon et al., 2013). In contrast, the ML models used in this study adopt a data-driven and prediction-oriented framework with minimal reliance on predefined pharmacokinetic structures. ML model development primarily involves algorithm selection and hyperparameter tuning, offering greater computational and practical efficiency, albeit at the cost of reduced direct biological interpretability and increased dependence on data quality (Keutzer et al., 2022).

Consistent with these differences, one previous study reported that all tested ML algorithms substantially outperformed population pharmacokinetic models in terms of pure computational run time, with ML models being at least 22 times faster per run (Keutzer et al., 2022). Because population pharmacokinetic model development requires repeated executions, these run time results were used to estimate overall development time, revealing a substantial cumulative computational burden. In contrast, many ML algorithms incorporated embedded feature selection and generally required only a single model run, in addition to hyperparameter tuning. Accordingly, although total model development time was not directly measured, the study suggested that both cumulative run time and overall development timelines were considerably shorter for ML approaches, whereas population pharmacokinetic model development could require several weeks even for experienced modelers (Keutzer et al., 2022).

Despite these advantages, population pharmacokinetic modeling remains uniquely suited for physiologically meaningful interpretation and for simulation of clinical scenarios not explicitly represented in the original dataset, such as dose optimization in special populations (Keutzer et al., 2022). Accordingly, ML should be regarded not as a replacement for population pharmacokinetics, but as a complementary approach for pharmacokinetic prediction. In the present study, the proposed ML model can be used to support dose optimization for IV infliximab given at 8-week intervals by simulating conditional concentration predictions based on prior drug levels to identify doses that achieve target concentrations.

In both infliximab and ADA prediction tasks, tree-based models such as RF and XGBoost consistently outperformed RNN-based models. Similar trends have been observed for other drugs in previous studies, further supporting the generalizability of tree-based algorithms in drug concentration prediction. For instance, Huang et al. demonstrated that the XGBoost model outperformed other ML models and population pharmacokinetic approaches in predicting voriconazole trough concentrations (Huang et al., 2025). Likewise, Tang et al. reported that Categorical Boosting (CatBoost), a tree-based model similar to XGBoost, achieved superior performance to population pharmacokinetic modeling in predicting isoniazid concentrations and determining appropriate dosing (Tang et al., 2024). This may be attributed to the fact that tree-based algorithms are well-suited for capturing complex non-linear interactions among clinical and laboratory covariates, even in the absence of explicit temporal structures. In contrast, RNN-based models like LSTM and GRU are designed to leverage sequential dependencies, which may not have been sufficiently strong or informative in this dataset to provide a clear advantage.

Moreover, in RNN-based models, we observed signs of temporal prediction lag, a well-documented limitation of RNN (Zhang et al., 2024). This refers to the model’s inability to promptly reflect abrupt changes or real-time variations in drug concentrations, resulting in a delayed output response. Such lag is particularly pronounced when time-series data are sparse or irregular, as was the case in our study. To mitigate this issue, we implemented several established techniques—DILATE (DIstortion Loss including shApe and TimE) loss function (Le Guen and Thome, 2019). and two decomposition techniques (empirical mode decomposition (Huang et al., 1998) and variational mode decomposition (Zosso et al., 2014))—but these efforts did not lead to meaningful improvements in prediction accuracy. This persistent lag likely contributed to the suboptimal performance of RNN-based models compared to tree-based algorithms.

In the multi-step prediction task, the models exhibited distinct patterns in performance between infliximab and ADA concentration forecasting. For infliximab, prediction accuracy declined progressively with each recursive step, with a marked deterioration observed at Step 3. Indeed, over the course of 8-week cycle, infliximab levels fluctuated significantly, gradually decreasing from maximal concentration after infusion, the rapidity of the decline being dictated by the rate of clearance in the individual (Gibson et al., 2020). Because of such conditions, using of trough level is theoretically the best time to guarantee that adequate exposure of infliximab was maintained across the time interval. This sharp drop likely reflects the compound effects of recursive error propagation, where inaccuracies from prior predictions accumulate and amplify over time. Moreover, given that infliximab concentrations are influenced by complex and dynamic factors, such as immune responses, concomitant medications, and inflammatory activity, predicting long-term trajectories becomes increasingly difficult without updated input data (Hemperly and Vande Casteele, 2018). Considering relatively stable level of subcutaneous infliximab levels throughout two-weak cycle, multi-step prediction task with subcutaneous infliximab might be more accurate and suitable for prediction. However, further study is needed. In contrast, the ADA prediction model maintained stable performance across all three steps. Even at Step 3, key evaluation metrics, including F20 (57.14%), F30 (71.43%), and 2-fold accuracy (96.43%), remained within acceptable ranges. This robustness may be attributed to the more gradual and predictable nature of ADA dynamics, as ADA levels, once formed, tend to change slowly over time and are highly correlated with prior measurements. This was why clinicians check once the ADA levels when ADA formation was suspected, such as loss of response in actual clinical practice. As such, ADA appears to follow a more stable temporal pattern, making it more amenable to recursive forecasting.

SHAP analysis provided meaningful insights into the relative contribution of each feature to model predictions, and the findings were largely consistent with established clinical and pharmacological knowledge. SHAP analysis confirmed that key predictors aligned with established pharmacological knowledge. In the prediction of infliximab concentration, past infliximab concentration was identified as the most influential feature, followed by the time interval since the last administration. These results are pharmacokinetically plausible, as shorter intervals between drug administration and serum sampling are associated with higher concentrations due to limited drug clearance. Similarly, an increased dose ratio, reflecting a higher administered dose compared to the previous dose, was associated with higher subsequent infliximab concentrations. Notably, the moderate contribution of inflammatory markers such as CRP suggests that disease activity may influence infliximab exposure beyond dosing history alone. This finding supports the clinical need for more frequent TDM in patients with fluctuating inflammatory status. Additionally, the contribution of past ADA levels in the infliximab model highlights the role of immunogenicity as a modifier of drug pharmacokinetics, supporting closer monitoring in patients with detectable ADA. In the ADA prediction model, past ADA concentration was the most significant predictor. The strong influence of past ADA levels is consistent with immunological understanding, as ADA formation tends to persist once it occurs and often remains stable or increases gradually over time. This temporal stability suggests that, during maintenance infliximab therapy, a single ADA measurement may be sufficient to inform future ADA status in many patients.

The proposed models demonstrate potential clinical utility in supporting personalized therapeutic strategies for patients who are treated with IV infliximab. By predicting more accurate future infliximab and ADA concentrations using ML models, clinicians may adjust dosing regimens and monitoring intervals more precisely. This approach could help optimize drug exposure, minimize immunogenicity, and reduce the likelihood of treatment failure. Importantly, the model enables prediction of infliximab concentrations up to two future time points based on a single serum sample, which could significantly reduce the frequency of TDM. This capability not only has the potential to lower the financial burden associated with repeated drug level testing, but also to improve patient convenience by reducing the need for frequent blood draws.

This study has several limitations. First, the analysis was based on prospectively collected data from a single tertiary care center, which may limit the generalizability of the findings. External validation using multicenter datasets is necessary to confirm the robustness and applicability of the models across diverse patient populations and healthcare environments. In addition, the model was developed in a selected population of patients with IBD receiving maintenance IV infliximab therapy. Therefore, the findings may not be directly applicable to patients with intermittent or recently interrupted infliximab treatment or those undergoing induction or switching between IV and subcutaneous formulations. Second, important external factors that change over time, such as concomitant immunomodulator therapy, and disease activity were not fully captured in the current dataset, although they may significantly influence infliximab pharmacokinetics and immunogenicity (Hemperly and Vande Casteele, 2018; Fasanmade et al., 2011; Vande Casteele et al., 2018). This limitation may particularly affect the performance of recursive multi-step forecasting, as temporal fluctuations in disease activity and treatment context cannot be fully reflected. Future research could incorporate these variables to improve model accuracy. Third, the model’s predictive performance is inherently limited to the scope of the input features used for training. In particular, SHAP analysis indicated a strong reliance on prior infliximab and ADA concentrations, suggesting reduced predictive performance when recent drug or antibody measurements are unavailable. Lastly, the models were developed to predict trough infliximab concentrations rather than area under the concentration–time curve. Therefore, their utility in estimating systemic exposure is limited.

## 5 Conclusion

This study developed ML models to predict infliximab and ADA concentrations in IBD patients receiving maintenance IV infliximab therapy, with RF and XGBoost showing the best performance for infliximab and ADA prediction, respectively. The ADA prediction model showed stable performance across three recursive steps, while infliximab prediction maintained acceptable accuracy up to two forecasting steps. These models may support individualized dosing strategies and reduce the need for frequent TDM in clinical practice.

## Data Availability

The raw data supporting the conclusions of this article will be made available by the authors, without undue reservation.
