# Supplementary material for: Prediction of infliximab and anti-drug antibody concentrations in patients with inflammatory bowel disease using machine learning models with real-world data from a prospective cohort study
Source: Front Pharmacol. 2026 Jan 28;17:1731193. doi: 10.3389/fphar.2026.1731193 (PMC12890613; doi:10.3389/fphar.2026.1731193)

Supplementary Table 1. Search spaces and selected values from hyperparameter tuning.

| Model | Hyperparameter | Search space | Best value |
| --- | --- | --- | --- |
| Elastic Net | L1_ratio | [0.1, 0.5, 0.9, 1.0] | 0.5 |
| Support Vector Regression | C | [0.1, 1, 10] | 10 |
|  | epsilon | [0.01, 0.1, 1] | 1 |
|  | kernel | rbf | rbf |
|  | gamma | [‘scale’, ‘auto’] | scale |
| Random Forest | n_estimators | [100, 200] | 100 |
|  | max_depth | [10, 20] | 20 |
|  | min_samples_split | [2, 5] | 5 |
|  | min_samples_leaf | [1, 2] | 2 |
| XGBoost | n_estimators | [100, 200] | 100 |
|  | max_depth | [3, 5, 7] | 7 |
|  | learning_rate | [0.05, 0.1] | 0.05 |
|  | subsample | [0.8, 1.0] | 0.8 |
|  | colsample_bytree | [0.8, 1.0] | 0.8 |

Supplementary Figure 1. Sequential data preprocessing using a sliding window approach for time-series prediction. In this method, the input features included the administered infliximab dose (Dose), the time interval between drug administration and serum sampling (Days), and the concentrations of infliximab and anti-drug antibody (ADA) from time points -3 to -1. These, along with the dose and sampling interval at time 0, are used to predict the infliximab and ADA concentrations at time 0.


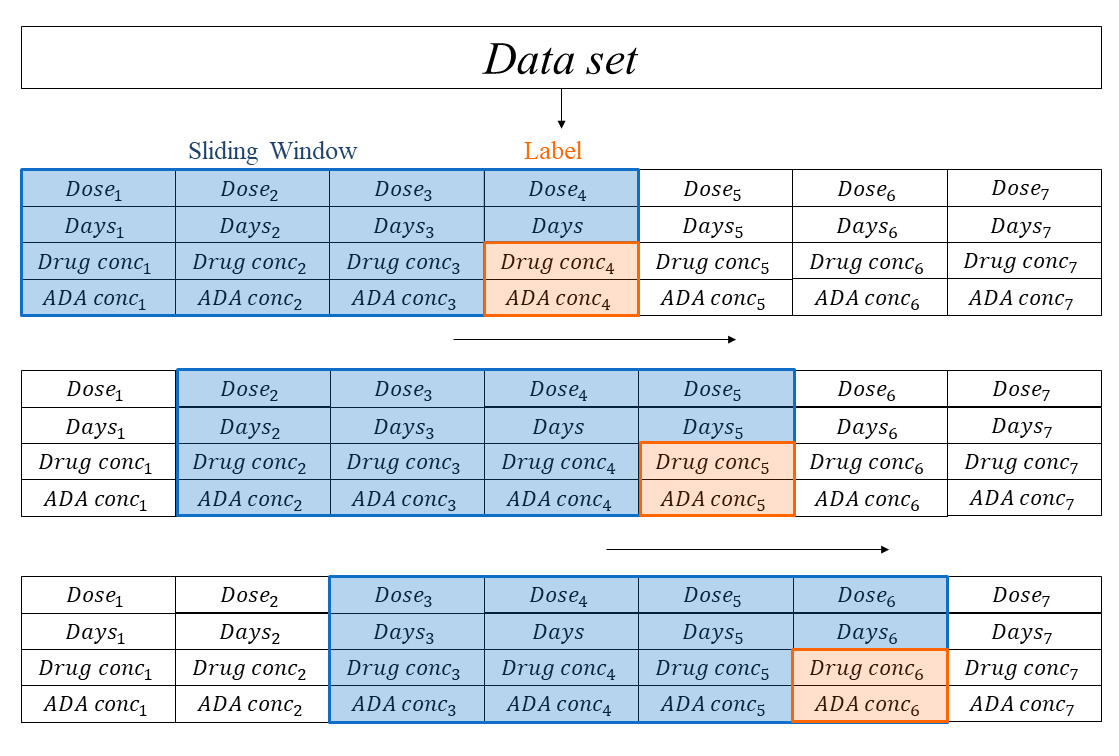


Supplementary Figure 2. Individual-level observed vs. predicted concentrations of (A) infliximab and (B) anti-drug antibody (ADA) across three recursive prediction steps (x-axis: time step 1–3). Line plots of observed (blue) and predicted (orange) concentrations for 15 randomly selected patients from the test set. Each subplot corresponds to an individual patient.


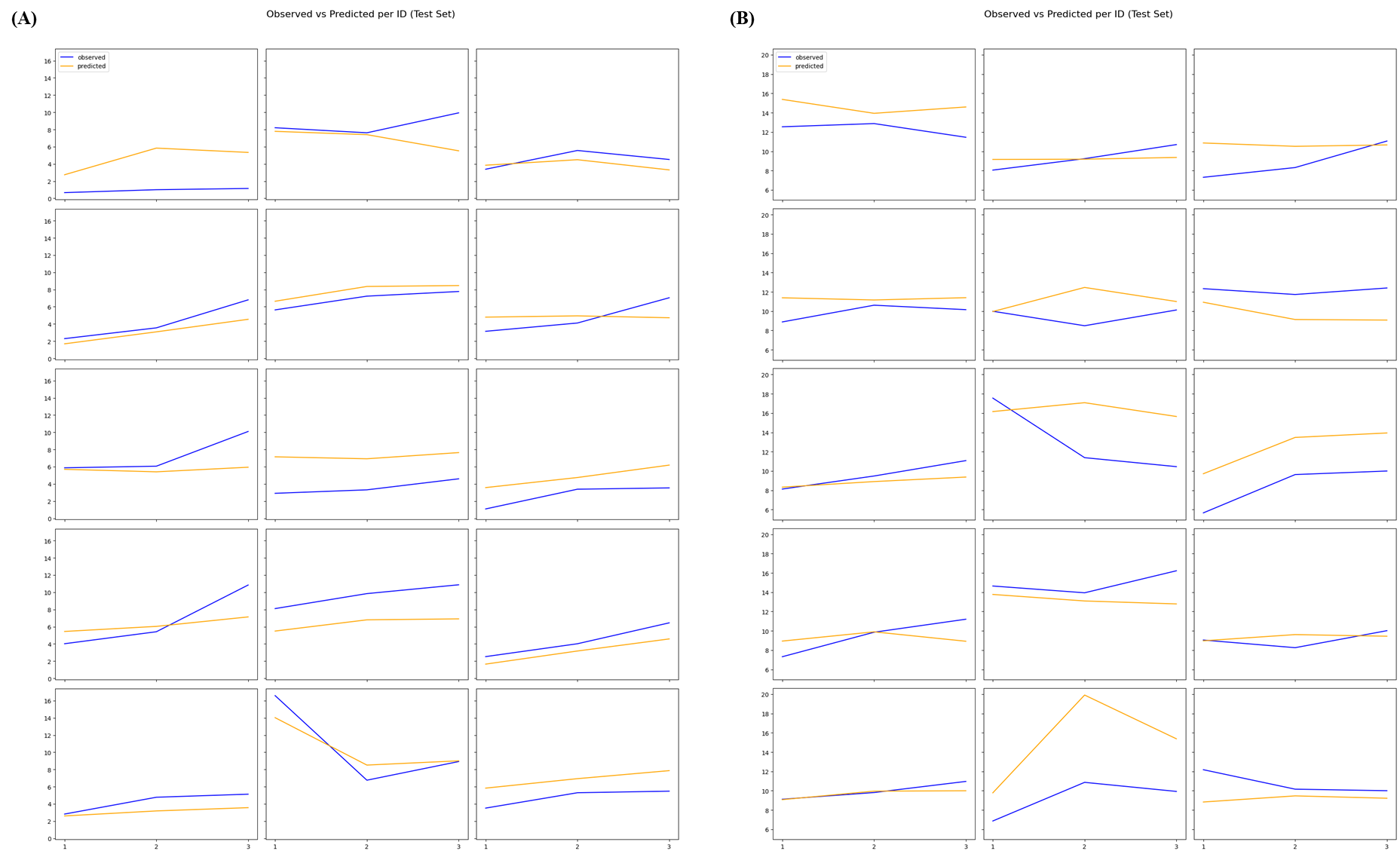

Supplement: Supplementary file 1 [file DataSheet1.docx]
